# *In silico* design and mechanistic study of niosome-encapsulated curcumin against multidrug-resistant *Staphylococcus aureus* biofilms

**DOI:** 10.3389/fmicb.2023.1277533

**Published:** 2023-11-30

**Authors:** Mohammad Khaleghian, Hamidreza Sahrayi, Yousef Hafezi, Mahshad Mirshafeeyan, Zahra Salehi Moghaddam, Bahareh Farasati Far, Hassan Noorbazargan, Amir Mirzaie, Qun Ren

**Affiliations:** ^1^Department of Chemistry, Payame Noor University, Tehran, Iran; ^2^Department of Chemical and Petrochemical Engineering, Sharif University of Technology, Tehran, Iran; ^3^School of Chemical Engineering, College of Engineering, University of Tehran, Tehran, Iran; ^4^Department of Microbial Biotechnology, School of Biology, College of Science, University of Tehran, Tehran, Iran; ^5^Department of Chemistry, Iran University of Science and Technology, Tehran, Iran; ^6^Department of Biotechnology, School of Advanced Technologies in Medicine, Shahid Beheshti University of Medical Sciences, Tehran, Iran; ^7^Department of Biology, Parand Branch, Islamic Azad University, Shahr-e Jadid-e Parand, Iran; ^8^Laboratory for Biointerfaces, Empa, Swiss Federal Laboratories for Materials Science and Technology, St. Gallen, Switzerland

**Keywords:** anti-biofilm, curcumin, niosome, *Staphylococcus aureus*, *in silico* studies, ADME prediction, molecular docking

## Abstract

Curcumin, an important natural component of turmeric, has been known for a long time for its antimicrobial properties. This study aimed to investigate the anti-biofilm action of the niosome-encapsulated curcumin and explore the involved anti-biofilm mechanism. *In silico* investigations of ADME-Tox (absorption, distribution, metabolism, excretion, and toxicity) were first performed to predict the suitability of curcumin for pharmaceutical application. Curcumin showed low toxicity but at the same time, low solubility and low stability, which, in turn, might reduce its antimicrobial activity. To overcome these intrinsic limitations, curcumin was encapsulated using a biocompatible niosome system, and an encapsulation efficiency of 97% was achieved. The synthesized curcumin-containing niosomes had a spherical morphology with an average diameter of 178 nm. The niosomal curcumin was capable of reducing multi-drug resistant (MDR) *Staphylococcus aureus* biofilm 2–4-fold compared with the free curcumin. The encapsulated curcumin also demonstrated no significant cytotoxicity on the human foreskin fibroblasts. To understand the interaction between curcumin and *S. aureus* biofilm, several biofilm-related genes were analyzed for their expression. N-acetylglucosaminyl transferase (*IcaD*), a protein involved in the production of polysaccharide intercellular adhesion and known to play a function in biofilm development, was found to be downregulated by niosomal curcumin and showed high binding affinity (-8.3 kcal/mol) with curcumin based on molecular docking analysis. Our study suggests that the niosome-encapsulated curcumin is a promising approach for the treatment of MDR *S. aureus* biofilm and can be extended to biofilms caused by other pathogens.

## 1 Introduction

Phytochemical components are critical sources in pharmaceutical applications. They can be used as new drug candidates for treating many diseases (Singh and Dutta, 2020). Considering that safe and alternative antibacterial agents are needed to decrease antibiotic-resistant bacteria (Kumbar et al., 2020), natural antimicrobial agents originated from herbal extracts are of great interest. Among natural products, curcumin has outstanding potential in antibacterial fields (Gagandeep et al., 2020). Although studies have shown significant antimicrobial effects of curcumin, some of its inherent physicochemical properties, including low solubility in water, have limited its use. Curcumin has thus been formulated for effective delivery, including in the form of niosome (Moeini et al., 2020).

Niosomes are biocompatible bilayered structures made of non-ionic surfactants and cholesterol (as a lipid) (Sharifi et al., 2020). These water-soluble nanoparticles are highly biocompatible and can carry both hydrophilic and hydrophobic drugs (Moosavian et al., 2020). This type of drug delivery system protects against the destruction of drugs in adverse environmental conditions and chemical degradation and increases the physical stability of the drug (Sapkota et al., 2019; Hojabri et al., 2023). Various niosomal formulations have been developed for a variety of drug delivery systems (Moghtaderi et al., 2021). The optimization of size and drug encapsulation efficiency has been performed to gain small-size niosomes with higher encapsulation efficiency for any particular drug (Mansouri et al., 2021). Several studies showed that curcumin encapsulated in nanostructures has greater therapeutic efficiencies than free curcumin (Gugleva et al., 2019). A niosomal curcumin system consisting of non-ionic surfactants such as Span 80, Tween 80, and Poloxamer 188 has been reported to have an encapsulation efficiency of 92.3%, allowing a controlled release of curcumin (Xu et al., 2016). Some curcuminoids (curcumin, desmethoxycurcumin, and bisdemethoxycurcumin) were also encapsulated into niosomes with an encapsulation efficiency of 83% (Rungphanichkul et al., 2011) and 95% (Akbari et al., 2020), achieving improved efficiency of transdermal delivery. However, it is not known yet whether the encapsulated curcumins can act against bacterial biofilms, which have been recognized to be involved in many clinical infections. *Staphylococcus aureus*, one of the most prevalent contagious pathogens, can cause several diseases from skin infections to fatal ones such as pneumonia (Bezerra Filho et al., 2020). *S. aureus* shows increased occurrence of antibiotic resistance resulting in emerging multidrug-resistant strains (Sharifi et al., 2021). Multidrug-resistant (MDR) *S. aureus* causes many opportunistic nosocomial infections and has made treatment difficult due to the mechanisms of antimicrobial resistance and the ability to form biofilms in living and non-living surfaces, especially medical implants (Roy and Rhim, 2020). Studies show that the intercellular adhesion (*ica*) operon, among others, is responsible for the production of biofilm; thus inhibition of *ica* expression can lead to a decrease in biofilm formation (Fitzpatrick et al., 2005). Recently, researchers have been looking for new ways to find new compounds with anti-biofilm properties, including several chemicals repressing *ica* transcription (Park et al., 2005). Molecular docking has been used to mimic the biological system and predict the binding affinity and binding conformation of a given molecule with the target (Mohammad et al., 2021).

In this study, the main goal is to develop niosome-encapsulated curcumin nanoparticles to fight against multidrug-resistant *S. aureus* and its biofilm formation. Furthermore, *in silico* analysis was used as a powerful tool to evaluate the pharmacokinetic/pharmacodynamic properties of curcumin and molecular docking to predict the performance of curcumin as an agent to inhibit biofilm formation in our biological system. The predicted interactions between curcumin and biofilm were confirmed by empirical studies by examining the anti-biofilm effects of curcumin-containing niosome and its effect on *icaD* biofilm gene expression in MDR *S. aureus* strains. The obtained *in silico* information not only revealed the drawbacks of curcumin but also helped to introduce an appropriate drug delivery system.

## 2 Materials and methods

### 2.1 Materials

Chloroform, ethanol, Span 80, diacetyl phosphate (DCP), DMSO, cholesterol, SDS, and Amicon (Ultra-15-Membrane, MWCO 30 kDa) were purchased from Merck company, Germany. RPMI-1640 Medium, DMEM, PBS, FBS, MTT, and penicillin/streptomycin 100 X were purchased from Gibco, USA. Dialysis membrane (MWCO 12 kDa) were purchased from Sigma-Aldrich, USA. All microbiological materials including culture media and chemicals were purchased from Sigma–Aldrich, USA. *Staphylococcus aureus* ATCC 700698 as a control strain was purchased from the American Type Culture Collection (ATCC). In addition, the HFF normal cell line was the cell bank of the Pasteur Institute of Iran. Curcumin (purity >96%) was obtained from Bio Basic Inc. (Canada). All molecular kits were purchased from Fermentas, Lithuania.

### 2.2 Box–Behnken design

To evaluate the effect of independent variables (drug concentration, molar ratio of surfactant to cholesterol, and molar ratio of lipid to cholesterol), the Box–Behnken model was used with Design-Expert 10.0.3 software (Stat-Ease Inc., USA). In addition, the effect of these variables on particle size and encapsulation efficiency (EE) was examined. The optimal formula was selected based on the minimum size of niosomes and the maximum amount of encapsulation efficiency. These agents and their levels are presented in Supplementary Table S1. The usefulness index was considered in optimizing D-optimal design data (Dwivedi et al., 2019). Finally, the optimal formula was selected for further studies (Gugleva et al., 2019).

### 2.3 Preparation of niosome

**Scheme 1:**
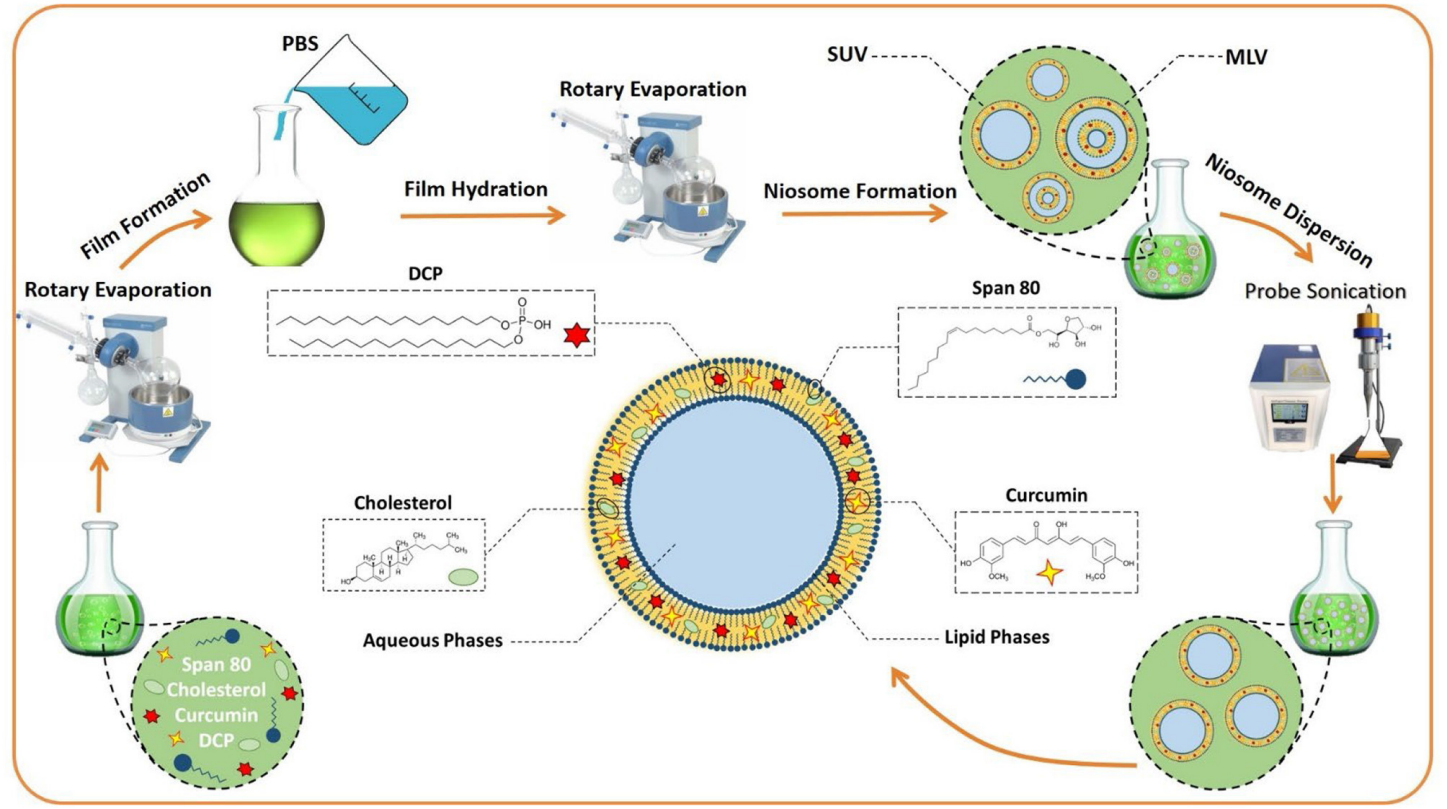
The preparation of niosomes by the thin layer hydration method. MLV, multilamellar vesicles; SUV, Small unilamellar vesicles; DCP, diacetyl phosphate; PBS, Phosphate-buffered saline.

The thin-layer hydration procedure was used for the preparation of curcumin-encapsulated niosome detailed in our published article with some modifications (Akbarzadeh et al., 2021) (Scheme 1). In brief, 10 mg of curcumin was mixed with lipids (1:12 molar ratio of drug to lipid), and the mixture was dissolved in 10 ml of 1:1 molar ratio of chloroform/methanol. The lipid contained a 1:1 molar ratio of surfactant to cholesterol, and the surfactant was a mixture of Span 80 and diacetyl phosphate (DCP) with a 1:1 molar ratio. The final mixture had 10 mg/ml curcumin, which was then evaporated using the rotary evaporator for 30 min at 60°C and 150 rpm. Afterward, the dried thin films were hydrated using 10 ml PBS at 30°C for 1 h with stirring at 120 rpm. Subsequently, the samples were sonicated (Hielscher UP50H ultrasonic processor, Germany) for 5 min and stored at 4°C for further usage. All of the concentrations and amounts of the drug and other components used in the final formulation were first optimized; the details of the process are explained in the Supporting File and the following.

### 2.4 Characterization of the synthesized niosomes

The morphology of the prepared niosomes was examined using a scanning electron microscope (SEM) and a transmission electron microscope (TEM) (Zeiss EM900, Germany). Particle size (dynamic diameter of nanoparticles) and polydispersity index (PDI) were evaluated at room temperature using a benchtop dynamic light scattering system/electrophoretic light scattering system (Zetasizer Nano S90, Malvern Instrument, UK). Niosome formulations were appropriately diluted (1:100) with deionized water to avoid multiple scattering phenomena due to antiparticle interactions. All measurements were repeated in triplicates.

The molecular interaction between curcumin and niosomes was investigated by Fourier-transform infrared (FTIR) spectroscopy (Spectrum Two, USA). Lyophilized samples were combined with KBr, pressed into a pellet by positioning the samples in a hydraulic press, and scanned in the range of 4,000–400 cm^−1^ in a fixed resolution of 4 cm^−1^ at room temperature on a BRUKER FTIR model (VERTEX 70).

Differential scanning calorimetry (DSC) analysis was performed using a differential scan calorimeter (TA, Q600, USA) for niosome, curcumin, and curcumin-loaded niosome samples. X-ray diffraction (XRD) studies of curcumin, curcumin-loaded niosomes, and free niosomes were performed using X'Pert Pro MPD (PANalytical, the Netherlands) by placing the samples in plane glasses. Small-angle XRD was acquired using an X-ray super-speed diffractometer with a Ni filter and Cu radiation (λ = 0.542 nm), tube voltage 25–45 kV, and tube current 100–200 mA, and scanned from 2° to 80°, 2θ.

### 2.5 Encapsulation efficiency (EE) and stability studies

The suspension containing curcumin-loaded niosomes was filtered using the Amicon Ultra-15 membrane (MWCO 30 kDa) for 20 min at 4,000 g. During filtration, the niosomes remained in the upper chamber, and free curcumin was removed through the filter membrane. Curcumin concentration was calculated by drawing a standard curve and reading the UV absorbance at 420 nm (Supplementary Figure S1). The encapsulation efficiency was studied using the following formula:


(1) $$\begin{array}{l} Encapsulation\;Efficiency\;\left(\%\right)=\left[\left(A-B\right)/A\right]\times \;100 \end{array}$$

A: the amount of the primary drug used in niosome synthesis, B: the amount of free drug that passes through the filter membrane.

For the stability studies, niosome formulations were stored at 25°C and 4°C for 2 months, and the nanoparticle size, PDI, and EE at specified intervals (0, 14, 30, and 60 days) were evaluated.

### 2.6 Drug release study

The *in vitro* drug release scrutiny was performed by putting 2 ml of each sample in a dialysis bag (MWCO of 12 kDa) that was placed in a 50 ml PBS solution at different pH (3, 5, and 7.4) and 37°C, accompanied by moderate stirring (50 rpm). Aliquots were sampled at certain time intervals and replaced by fresh PBS solution. The release profile was analyzed by adopting various release kinetic models (information on release kinetic models is presented in SI). A drug release test was also performed for the free drug with a similar initial drug concentration.

### 2.7 *In silico* analysis

*In silico* analysis focuses on the ADME-Tox (absorption, distribution, metabolism, excretion, and toxicity) of curcumin to predict any potential drawbacks. By understanding the ins and outs of the drug, we can select the best nanocarrier to resolve any problem. Additionally, molecular docking can predict the binding affinity, preferred orientation, and binding conformations between the selected genes in biofilm formation and curcumin. This can help us understand whether curcumin plays a crucial role in inhibiting these genes or not, thus preventing any experimental failures. The details of the procedure related to the *in silico* analysis are presented in the Supplementary material.

### 2.8 Microbiological studies

#### 2.8.1 The studied strains

Bacterial strains tested in this study are as follows: the isolated *S. aureus* strains; gram-positive strains *methicillin-resistant S. aureus* (*MRSA*) ATCC 700698, *S. epidermidis* ATCC 12228, *Bacillus subtilis* ATCC 21332, and *Streptococcus pyogenes* ATCC 14918; and gram-negative strains *Escherichia coli* ATCC 25922, *Pseudomonas aeruginosa* ATCC 27853, *Klebsiella pneumoniae* ATCC 700603, and *Proteus mirabilis* ATCC 12453.

#### 2.8.2 Isolation of *Staphylococcus aureus* strains

In this study, for the isolation of *S. aureus* strains, clinical samples such as urine, blood, sputum, and cerebrospinal fluid were collected for 3 months. *S. aureus* isolates were recovered from clinical samples using standard microbiological methods (Baron and Finegold, 1990). *S. aureus* isolates were kept at −80°C for subsequent steps.

#### 2.8.3 Antibiotic susceptibility test

Antibiotic susceptibility test was performed based on the Clinical Laboratory Standard Institute (CLSI) recommendation for the following antibiotics μg per disk: chloramphenicol (30), amoxicillin (10 μg), amikacin (15), gentamicin (10 μg), oxacillin (1), clindamycin (2), trimethoprim (25), erythromycin (15), penicillin (10), cefoxitin (30), and vancomycin (10). The minimum inhibitory concentration (MIC) values were determined according to the CLSI procedure as follows: free or encapsulated curcumin with 0, 15.62, 31.25, 62.5, 125, 250, and 500 μg/ml was mixed with bacteria of the final concentration of 5 × 10^5^ CFU/ml. The mixture was incubated for 24 h at 37°C. Using a visible UV spectrophotometer, the absorption at a wavelength of 600 nm was measured. The MIC is defined as the lowest concentration of drug that can inhibit bacterial growth. Multidrug resistant (MDR) strains are defined as strains that are resistant to at least one antibiotic in three or more antimicrobial groups.

#### 2.8.4 Phenotypic detection of methicillin-resistant isolates

MRSA strains were isolated using the disk diffusion test by cefoxitin disk (30 μg, with inhibition zone lower than 21 mm = MRSA) and oxacillin disk (1 μg, with inhibition zone lower than 10 mm = MRSA). All tests were performed in triplicate, and the results were interpreted based on the CLSI guidelines (Shabani et al., 2019).

#### 2.8.5 Biofilm formation

Isolation of biofilm-forming MDR-MRSA strains was performed using the 96-well microtiter plate method, as reported previously (Mohanta et al., 2020). In brief, MDR-MRSA strains were cultured in a TSB medium containing glucose (1% W/V) for 24 h. Then, 0.5 Mc Farland of each isolate was poured into a 96-well plate, followed by incubation for 24 h at 37 °C. The plate was then washed three times with PBS to remove the suspended bacterial cells. The biofilm biomass was quantified using crystal violet staining (Stiefel et al., 2016).

#### 2.8.6 Biofilm inhibition assay

The biofilm inhibitory potential of niosomal formulations was evaluated using a crystal violet (CV) assay. In brief, 180 μl of Müller Hinton Broth (MHB) culture medium and 20 μl of bacteria (10^7^ CFU/ml) were added to each well. In total, 100 μl of formulations with half of the MIC concentrations (Supplementary Table S2) were then added. The plate was incubated at 37°C for 24 h. Afterward, the supernatants were removed, and the wells were washed with PBS to remove the floating and non-adherent cells. The plates were then air-dried, fixed with 2% sodium acetate, and stained with 0.1% crystal violet in the dark for 30 min. The wells were then washed with PBS to remove excess dye. Finally, 200 μl of ethanol was added to the wells to elute crystal violet. The absorption at 590 nm was read using an ELISA reader (Stat Fax 2100, Awareness Tech Inc., USA).

#### 2.8.7 Gene expression analysis

Gene expression of the biofilm-related genes *icaA, icaB, icaC, icaD, fnbA*, and *clfA* and the quorum sensing genes *agrA, agrB*, and *agrC* in MDR-MRSA strains treated with half-MIC concentration of free curcumin and niosome encapsulated curcumin (Supplementary Table S2) was investigated using the quantitative real-time PCR method (Atshan et al., 2012; Srivastava et al., 2014; Bimanand et al., 2018; Tan et al., 2022). The qRT-PCR assay was performed using SYBER Green-containing Master Mix (Ampriqon, Denmark).

### 2.9 Cell toxicity assay

Cytotoxicity of the free curcumin, free niosome, and niosome-encapsulated curcumin was evaluated, employing normal human foreskin fibroblasts (HFF cell line, fibroblast, skin/temple, and SCRC-1041™). Nanoparticles (free niosome and niosome-encapsulated curcumin) and free curcumin of different concentrations ranging from 3.125 to 100 μg/ml were re-suspended with RPMI-1640 and then added to the HFF-seeded well (with 10,000 cells per well), which was further incubated for 24 h. A control (medium only) was used. After 24 h of incubation, 100 μl of MTT dye was poured into the wells, and the microplates were incubated at 37°C for 4 h. 100 μl of DMSO was added and the absorption were read at 570 nm. Finally, the IC90 samples were calculated.

### 2.10 Data analysis

In this study, all tests were performed three times, and the results were shown as mean standard deviation (SD) of one set of experimental data, and a one-way ANOVA test was used for the statistical analysis of data, and *p* < 0.05 was considered significant.

## 3 Results and discussion

### 3.1 ADME analysis of the suitability of curcumin as a drug

Inappropriate absorption, distribution, metabolism, and exertion characteristics (ADME) are imperative issues for drug application. Therefore, knowing more about the pharmacokinetics and pharmacodynamics features of curcumin can help to overcome these drawbacks. Here, 24 ADME features were checked based on the ADMETLAB standards, and the results are presented in SI (Supplementary Figure S2).

Overall, *in silico* features of curcumin include low bioavailability, poor water solubility (which leads to poor absorption), high degradation and metabolic rate, chemical instability, and short half-life when using it directly. To overcome these drawbacks and improve the therapeutic ability of curcumin, we fabricated and optimized a new drug delivery system.

### 3.2 Encapsulation of curcumin

**Figure 1:**
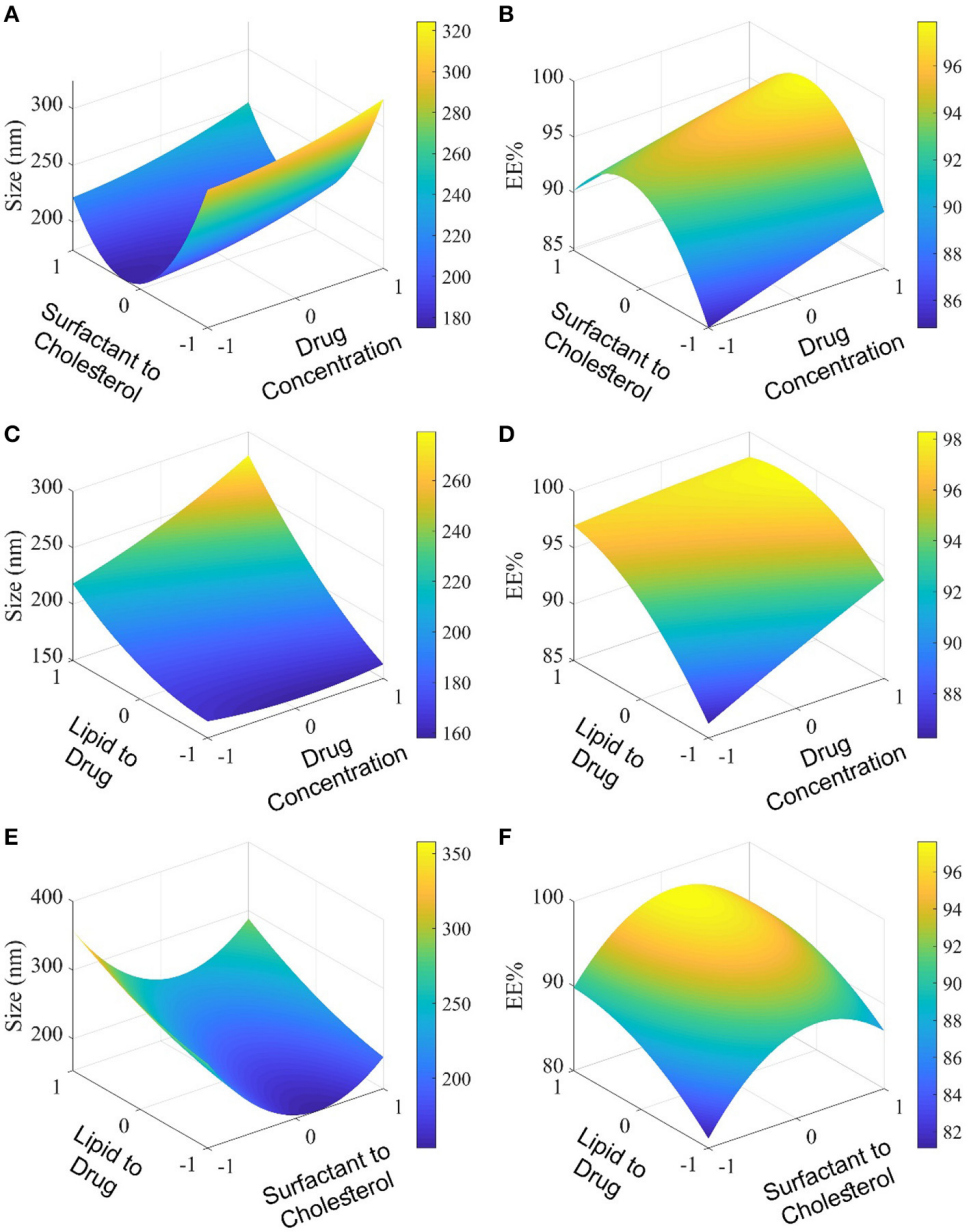
Evaluation of the molar ratio of surfactant to cholesterol and molar ratio of lipid to the drug by the Box–Behnken method. In **(A, B)**, the level of lipid to drug molar ratio is 0; In **(C, D)**, the level of surfactant to cholesterol molar ratio, and in **(E, F)**, the level of drug concentration is 0.

Niosome was used in this study to encapsulate curcumin. Independent variables such as curcumin drug concentration (A), surfactant (Span 80): cholesterol molar ratio (B), and lipid (cholesterol, DCP, and Span 80): drug molar ratio (C) were investigated with the Box–Behnken experimental design (Supplementary Table S1). The effects of these variables on responses (particle size and encapsulation efficiency) are presented in Supplementary Table S3 and Figure 1. The size and polydispersity index (PDI) can be found to vary in different niosomal formulations with different surfactants/cholesterol molar ratios, different lipid/drug molar ratios, and various drug concentrations.

Increasing the concentration of the drug and lipid: drug molar ratio led to an increased particle size of curcumin-loaded niosomes (Figure 1). However, the surfactant:cholesterol molar ratio showed an adverse effect on particle size. The encapsulation efficiency of curcumin-loaded niosomes was found in a narrow range from 80% to 97% (Supplementary Table S3, Figure 1). All the mentioned independent variables (drug concentration, surfactant:cholesterol molar ratio, and lipid:drug molar ratio) have positive and incremental effects on encapsulation efficiency. Analysis of variance and regression equation for particle size demonstrates that the response is polynomial, and the quadratic model is the best-fitted model with a *p* < 0.05 (Supplementary Table S4). The statistical analysis of encapsulation efficiency is presented in Supplementary Table S5, which shows that the quadratic model for this experimental data is significant. Moreover, the regression equation for encapsulation efficiency shows that the effect of independent variables such as A, B, and C, is incremental. The encapsulation efficiency of loaded niosomes is significantly affected by independent variables, such as A and C.

The optimized formulation based on prediction [drug concentration (1 mg/ml), surfactant:cholesterol molar ratio (1:1), and lipid:drug molar ratio (12:1), Supplementary Table S6] was successfully prepared with the particle size at 177±4 nm and EE at 97±1% (Supplementary Table S7).

It has been reported that the niosome size and encapsulation efficiency depend dramatically on the surfactant type and cholesterol amount (i.e., lipid) in the niosomal structure (Brahma et al., 2018). Previous studies reported that niosome formulations having Span 80 showed smaller sizes than other Spans. The reason for the phenomenon is due to the greater hydrophobic–hydrophobic interaction between encapsulated curcumin, DCP, cholesterol, and the hydrophobic chain in the structure of Span 80 (Das et al., 2016). In nanovesicular drug delivery systems, particle size is an important parameter that affects encapsulation efficiency and drug release (Bnyan et al., 2018). Our findings are in agreement with the previous studies, which showed that increasing the amount of cholesterol causes larger vesicles (Gowrishankar et al., 2016). This could be because cholesterol tends to increase the number of lipid bilayers (Chmiel et al., 2019). In contrast, the encapsulation efficiency decreased by increasing the cholesterol amount. Thus, optimization of the cholesterol ratio is necessary to obtain a high load of drugs from the niosomal structure (Park et al., 2005).

### 3.3 Characterization of niosome-encapsulated curcumin

**Figure 2:**
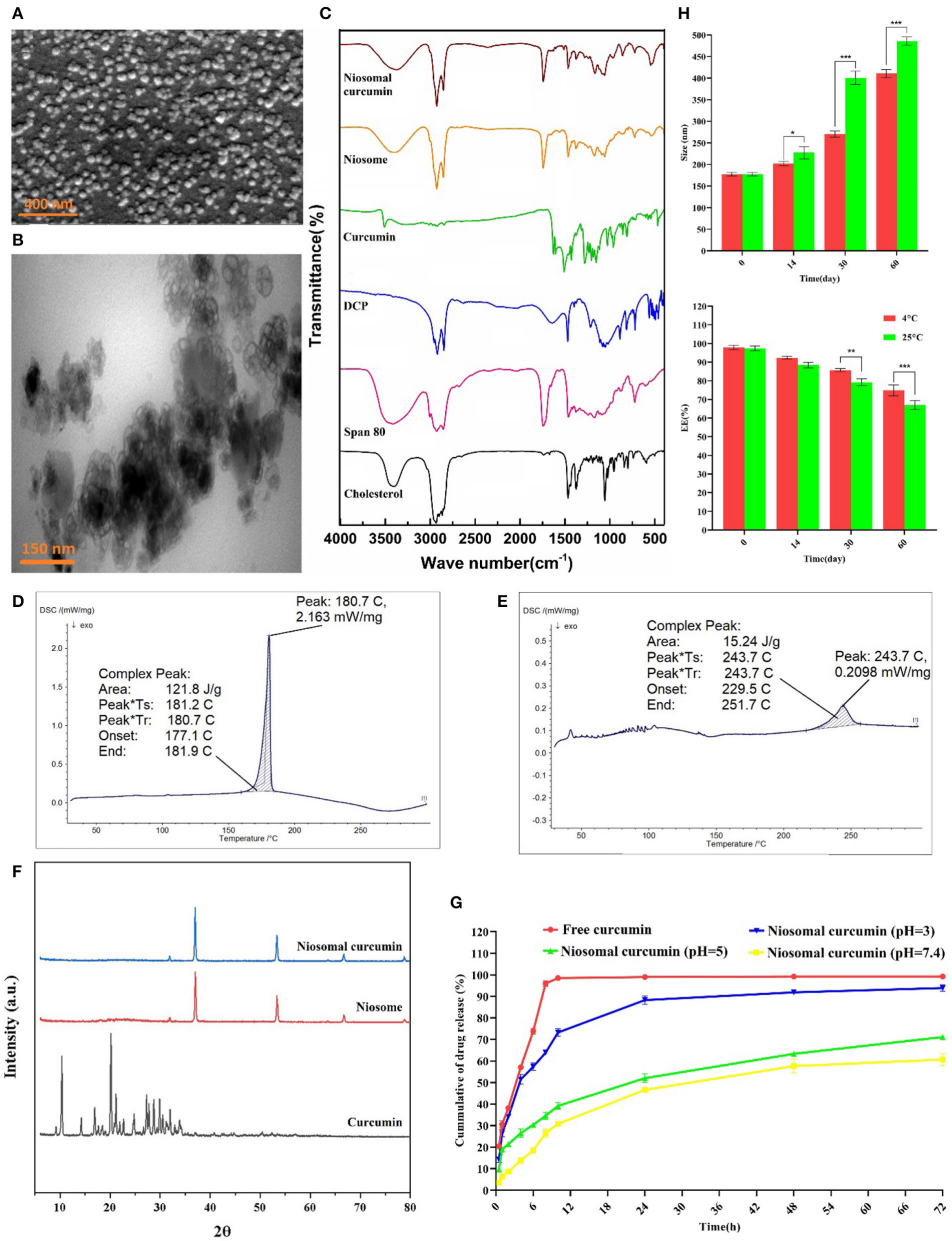
Morphological, structural, and stability determination of optimized formulation by **(A)** SEM, **(B)** TEM, **(C)** FTIR analysis, DSC spectrum of **(D)** Curcumin, and **(E)** Curcumin-loaded niosome, **(F)** XRD, **(G)** *In vitro* drug release of curcumin at different pH ranges, and **(H)** Stability of optimum curcumin-loaded niosomes (*n* = 3, *p* < 0.001***, *p* < 0.01**, and *p* < 0.05*).

The SEM and TEM results showed that the curcumin-loaded niosomes had a spherical structure with an average size of < 50 nm (Figures 2A, B). The particle size of the niosomes measured by the DLS method was approximately 178 nm. The size difference between the SEM and TEM and the DLS measurement could be caused by the drying process of the samples for the SEM and TEM imaging, while DLS measures the hydrodynamic diameter including the core with a molecule attached or adsorbed on the surface (Moghassemi et al., 2017).

### 3.4 Chemical, structural, and thermal analyses

According to the results obtained from FTIR, for the drug-free niosome, the spectra related to C–O–C bonding in Span 80, DCP, and cholesterol at 2,800–3,000 cm^−1^, 2,965 cm^−1^, and 1,125 cm^−1^ can be inferred, respectively (Figure 2C, Supplementary Table S8) (Akbari et al., 2020). The peaks at 1,674 cm^−1^ linking to C=C stretching in cholesterol were invisible in the FTIR spectra of niosomes, which indicates the encapsulation of cholesterol in the bilayer structure of the niosome (Bnyan et al., 2018). While the C-O-C bond of curcumin can be detected in free and niosomal curcumin, the main characteristic (aromatic ring, C=C stretching) summits of curcumin disappeared in the ultimate niosomal formulation, which has been reported previously (Di Francesco et al., 2017). The DSC spectrum of formulations showed a sharp endothermic peak at 187.7°C, which is related to curcumin melting point (Figure 2D). When curcumin is loaded into the niosome nanoparticles, the appeared peak shifts from 187.7°C to 243.7°C (Figure 2E), which is due to absorbing or loading curcumin into the niosome structure. Furthermore, the curcumin diffraction planes in the XRD analysis are not detected in the niosome-encapsulated curcumin sample (Figure 2F). Loading of the crystalline curcumin into the amorphous niosome, leading to the disappearance of curcumin peaks suggests the successful encapsulation of curcumin into niosomes. Some peaks in these structures are related to NaCl of the PBS solution used for suspending niosome (Supplementary Table S6). These results are consistent with the previous reports (Akbari et al., 2020).

### 3.5 *In vitro* drug release and kinetic models

Inspection of curcumin release from the optimized niosomal formulation was conducted for up to 72 h at different pH. As shown in Figure 2G, the drug release profile in the niosomal formulation is genuinely different from the free curcumin, likely because the restricted curcumin in the niosomal formulation should emerge from the lipid bilayer. In the first 8 h, the free curcumin was released up to 95%, while the encapsulated curcumin was released only 26%, 35%, and 64% at pH 7.4, 5, and 3, respectively, preventing burst release. After 72 h, the free curcumin was 100% released, while 61%, 71%, and 94% of the niosomal curcumin were released at pH 7.4, 5, and 3, respectively. The curcumin release profile declared that the cumulative release profile, in contrast to the free drug, is biphasic and limited (Sadeghi et al., 2019). The initial phase of rapid drug release is a uniform release mode, which may occur due to the release from the niosome surface. The slow release phase may be due to the release of curcumin from the bilayer structure of the niosome (Barani et al., 2018). Hence, the acidic environment could swell/break the niosomal formulation and significantly raise the curcumin release rate due to the hydrolysis of Span 80 in the niosome structure, and it can be applied in a pH-dependent drug delivery system (Hajizadeh et al., 2019).

Different release kinetic models were further examined at a certain pH to confirm the most fitting equation (see the additional data for each model in **Supplementary-Kinetic models**), which was also reported previously (Sadeghi et al., 2020). Analysis of curcumin release using Newsom showed that the Korsmeyer–Peppas model was the fittest (Supplementary Table S9) (Alemi et al., 2018). It appears that the release kinetics of curcumin from the optimum synthesized niosome at pH = 7.4 (*n* < 0.45: Fickian diffusion release mechanism) and acidic conditions (*n* > 0.45: non-Fickian diffusion mechanism) can be best described by the Korsmeyer–Peppa kinetic model (Park et al., 2005).

### 3.6 Physical stability of niosome-encapsulated curcumin

Steric/repulsion forces do not merely define the stability of niosomal formulations. It has been outlined that in the course of the storage process, the niosomes could swell/break down, which causes water molecules to diffuse into the niosomes. Therefore, the stability of the curcumin-loaded niosome was investigated during storage at two temperatures (25°C and 4°C) by measuring the size and encapsulation efficiency of the optimal formula. Figure 2H shows that with the increasing storage time, the sample size increased. Formulations stored at 4°C were more stable than those at 25°C, possibly due to the higher stiffness of the hydrophobic part of the niosome at lower temperatures. The storage of the drug in the niosomal formulation caused the drug to leak < 20% of the initial encapsulated amount in both conditions. These results are consistent with the findings of other research studies (Bnyan et al., 2018), which showed that storage at lower temperatures enhanced the shelf-life of niosome. Another reason for the lower stability at higher temperatures is the possibility that more water molecules can be diffused into the niosomal formulation, as the diffusion coefficient is in correlation with the temperature (Moghddam et al., 2016). The optimization of the cholesterol content in the formulations can increase the niosomal stability through interaction with the niosome membrane (Das et al., 2016).

### 3.7 Antibacterial activity and anti-biofilm formation

**Figure 3:**
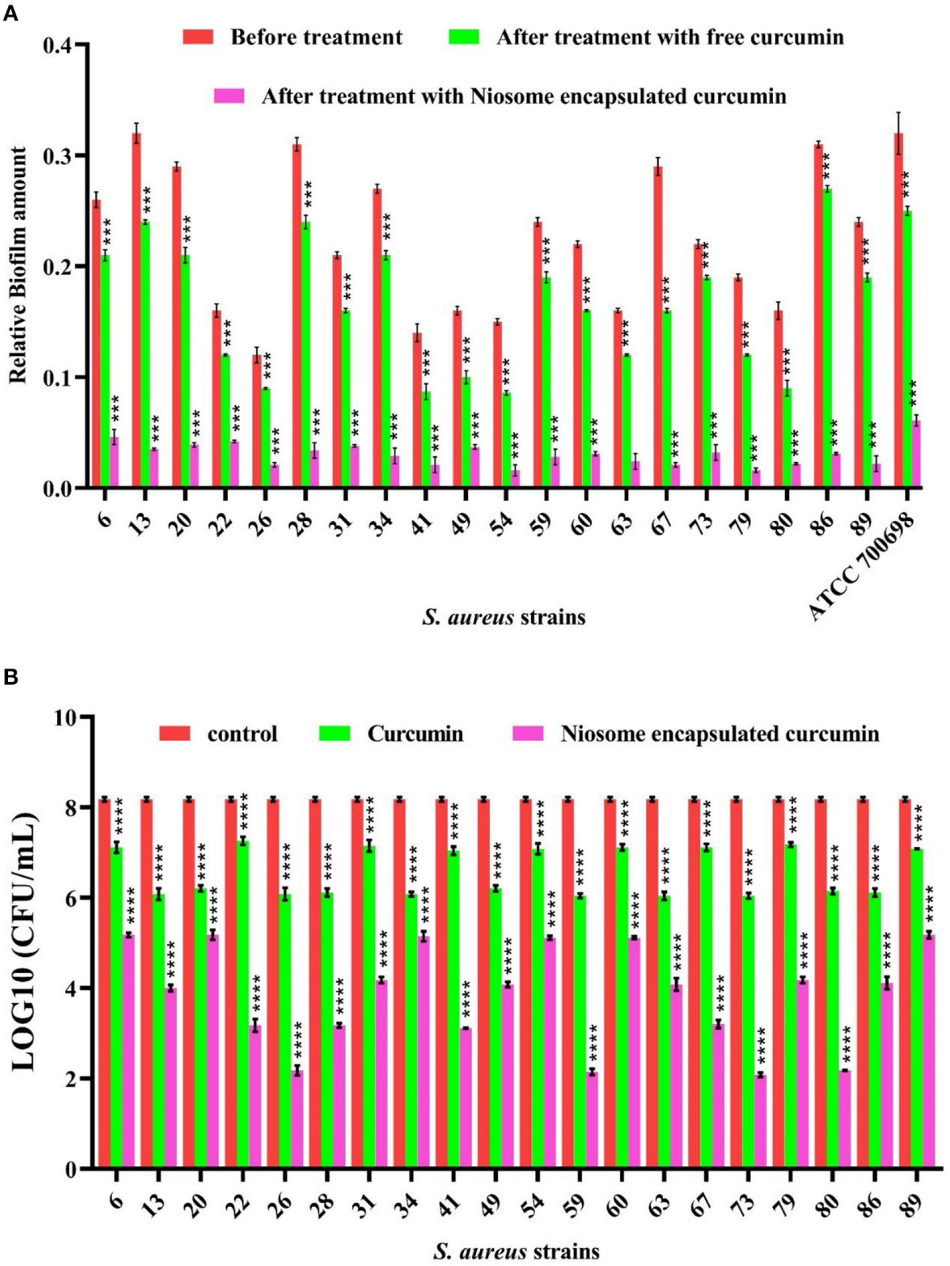
Anti-biofilm activity of free curcumin and niosome-encapsulated curcumin against MRSA measured by **(A)** crystal violet methods for total biofilm biomass and **(B)** CFU counting for viable bacteria. The untreated samples were used as controls (*n* = 3, *p* < 0.0001****, *p* < 0.001***, *p* < 0.01**, and *p* < 0.05*).

30 clinical isolates were analyzed for their antibiotic susceptibility and ability to form biofilm based on the crystal violet (CV) assay. In total, 20 isolates were found to be MDR and biofilm formers (Supplementary Table S10). MIC values were further measured to confirm the obtained results. The mean MIC values of free curcumin against these MDR methicillin-resistant *S. aureus* (MRSA) strains were 125 and 250 μg/ml, while the mean MIC values of niosomal-encapsulated curcumin against these MDR methicillin-resistant *S. aureus* (MRSA) strains were measured to be 15.62, 31.25, and 62.5 μg/ml (Supplementary Table S2). The MIC values of niosomal-encapsulated curcumin against all tested strains were significantly lower in comparison to free curcumin. These biofilm-forming MDR-MRSA strains were exposed to half-MIC of curcumin and niosome-encapsulated curcumin identified in this study (for MIC values of each strain, see Supplementary Table S2). The results of the CV test revealed that niosome-encapsulated curcumin significantly reduced MRSA biofilm biomass compared with free curcumin (Figure 3A). To evaluate whether the niosomal curcumin can kill bacterial cells in biofilm, the viability of biofilm cells was quantified using CFU counts. It was found that free curcumin caused a ~1–2 log decrease in viable cell count at the sub-MIC for the tested MDR-MRSA strains, while niosome-encapsulated curcumin led to a ~3–6 log reduction (Figure 3B). Indeed, the formulated niosome with half-MIC was also capable of eliminating other representative gram-negative and gram-positive bacterial species (Supplementary Table S11). Among the mechanisms interactions of niosomes with bacterial cell wall can be mentioned through contact release, fusion, and absorption (Nakagawa et al., 2020). In this study, to increase the solubility of curcumin and investigate the anti-biofilm effects of curcumin, its niosomal form was used. The results showed that niosomal curcumin has significantly higher antimicrobial and anti-biofilm activities than the free curcumin, likely due to the possibility that the niosome can be fused with the membrane of the bacterial cells and cause easy diffusion of the curcumin into the bacterial cell, leading to the targeted release of the drug into the bacterial cell and enhanced antibacterial activity. It has been reported that the antimicrobial and anti-biofilm effects of curcumin–silver and curcumin–copper are far higher when encapsulated in the niosomes than free curcumin (Targhi et al., 2021).

### 3.8 Protein modeling and recognition of target properties

Several studies speculated that niosomes could be bound to the biofilm, enhancing the delivery of the loaded drug to the microbial cells (Lee and Wang, 2020). To address this, we further utilized *in silico* modeling to investigate this possibility. Molecular docking was utilized for prediction. Modeling valid protein structures as target molecules is reflected to be the most practical in molecular docking. Within modeling, homology modeling is efficient when the model protein (with a specified sequence and unknown structure) is associated with at least another protein with a specified similar sequence and structure (Jalily Hasani et al., 2017). However, when the sequence identity decreases, the homology model reliability reduces too. Therefore, target–template pairs with < 30% sequence identity require template-free modeling (*de novo* or *ab initio*) with Robetta and trRosetta (Davahli et al., 2020). *S. aureus* biofilm formation is strongly regulated by the expression of intracellular adhesion of a polysaccharide (PIA) controlled by the *icaADBC* operon (Cue et al., 2012). In addition, this pathogen can express different types of fibronectin A-binding proteins (*FnbA*) and aggregating factors A (*ClfA*), which are involved in the binding of bacterial cells to surfaces and the formation of biofilms (Speziale and Pietrocola, 2020). Many pathogenicity factors, such as biofilm formation, are under the control of the regulatory system of the helper gene for quorum measurement (*agr*). The agr system, including *AgrB, AgrC, AgrA*, and *AgrD*, plays an important role in pathogenesis by regulating virulence factors and biofilm formation in *S. aureus* (Tan et al., 2018).

Using a Swiss model server based on homology modeling, the following were constructed: *IcaA* with 5EJ1 PDB ID template and 23.31% sequence identity, *IcaB* with 4WCG PDB ID template and 28.57 % sequence identity, *IcaC* with 6IH1 PDB ID template and 13.04% sequence identity, *IcaD* with 7D8X PDB ID template and 13.85% sequence identity, *argB* with 2BTY PDB ID template and 38.85% sequence identity, *ArgC* with 2BTY PDB ID template and 38.85% sequence identity, *FnbA* with 4B5Z PDB ID template and 100% sequence identity, *ClfA* with 1N67 PDB ID template and 100% sequence identity. Due to the lower sequence identities of built structures, the models (*IcaABCD, ArgB*, and *ArgC*) were not reliable, while *FnbA* and *ClfA* models were admitted with 100% sequence identity. Robetta and trRosetta servers have provided five models individually for *IcaABCD, ArgB*, and *ArgC*. Evaluation of TM-scores chosen trRosetta models as the best models against Robetta-predicted structures. Furthermore, using the trRosetta server, models 3, one, 5, 3, 3, and two were selected for *IcaA* (TM-score = 0.86), *IcaB* (TM-score = 0.85), *IcaC* (TM-score = 0.77), *IcaD* (TM-score = 0.50), *ArgB* (TM-score = 0.92), and *ArgC* (TM-score = 0.88), respectively.

### 3.9 Validation and refinement of proteins

A significant issue in structural biology is the recognition of errors in models of protein structures (Jalily Hasani et al., 2017). Thus, we evaluated the protein model to ensure the protein structure in terms of the distribution of torsion angles, the atomic coordinates of the model, and examination of atom–atom interactions in the structure (Jalily Hasani et al., 2017) by Ramachandran plot, Z-score, and ERRAT score, respectively. The accuracy of the trRosetta and Robetta servers was therefore validated by the Ramachandran plot, ERRAT score, and Z-score. The scores of trRosetta structures as the best model for *IcaABCD* and *ArgBC* are presented in Table 1. A potential protein would be expected to have over 90% amino acid residues in favorable regions. Moreover, no amino acid residue was presented in the disallowed and generosity-allowed regions. *IcaB* and *ArgB* have these features. Furthermore, we used homology modeling of *ClfA* instead of trRosetta and Robetta modeling due to all of the ERRAT scores being lower than 40%, in addition to the Ramachandran plot showing errors. Moreover, in terms of *FnbA*, 8 and 13 residues are in the generosity allowed and disallowed regions, respectively. We refined *ClfA, FnbA, IcaA, IcaB*, and *IcaD* because of reducing residues in the generosity allowed and disallowed regions.

**Table 1:** Validation of proteins using Ramachandran plot, Z-score, and ERRAT score.

**Proteins**	**ERRAT score (%)**	**z-score**	**Ramachandran plot**			
			**Favored region**	**Additional allowed**	**Generosity allowed**	**Disallowed**
*IcaA*	97.43	−5.18	91.5%	7.2%	0.5%	0.5%
*IcaA* after refinement	95.89	Not calculated	95.6%	3.6%	0.5%	0.3%
*IcaB*	94.32	−8.52	90.3%	10.1%	1.0%	0.7%
*IcaB* after refinement	90.07	−8.66	94.1%	5.9%	0%	0%
*IcaC*	91.48	−7.81	91.1%	8.0%	0.3%	0%
*IcaD*	98.90	−1.20	92.6%	7.7%	0.3%	0.5%
*IcaD* after refinement	97.80	Not calculated	98.9%	1.1%	0%	0%
*ArgB*	98.37	−9.07	89.6%	16.1%	0%	0%
*ArgC*	92.01	−8.65	93.4%	11.9%	2.5%	0%
*FnbA* before refinement	94.29	−7.48	91.3%	7.2%	1.4%	0%
*FnbA* after refinement	97.75	−7.63	97.7%	5.1%	0.0%	0.4%
*ClfA* before refinement	97.75	−8.20	89.8%	8.8%	1.1%	0.4%
*ClfA* after refinement	95.66	−8.30	93.3%	6.0%	0.4%	0.4%

### 3.10 Molecular docking

**Figure 4:**
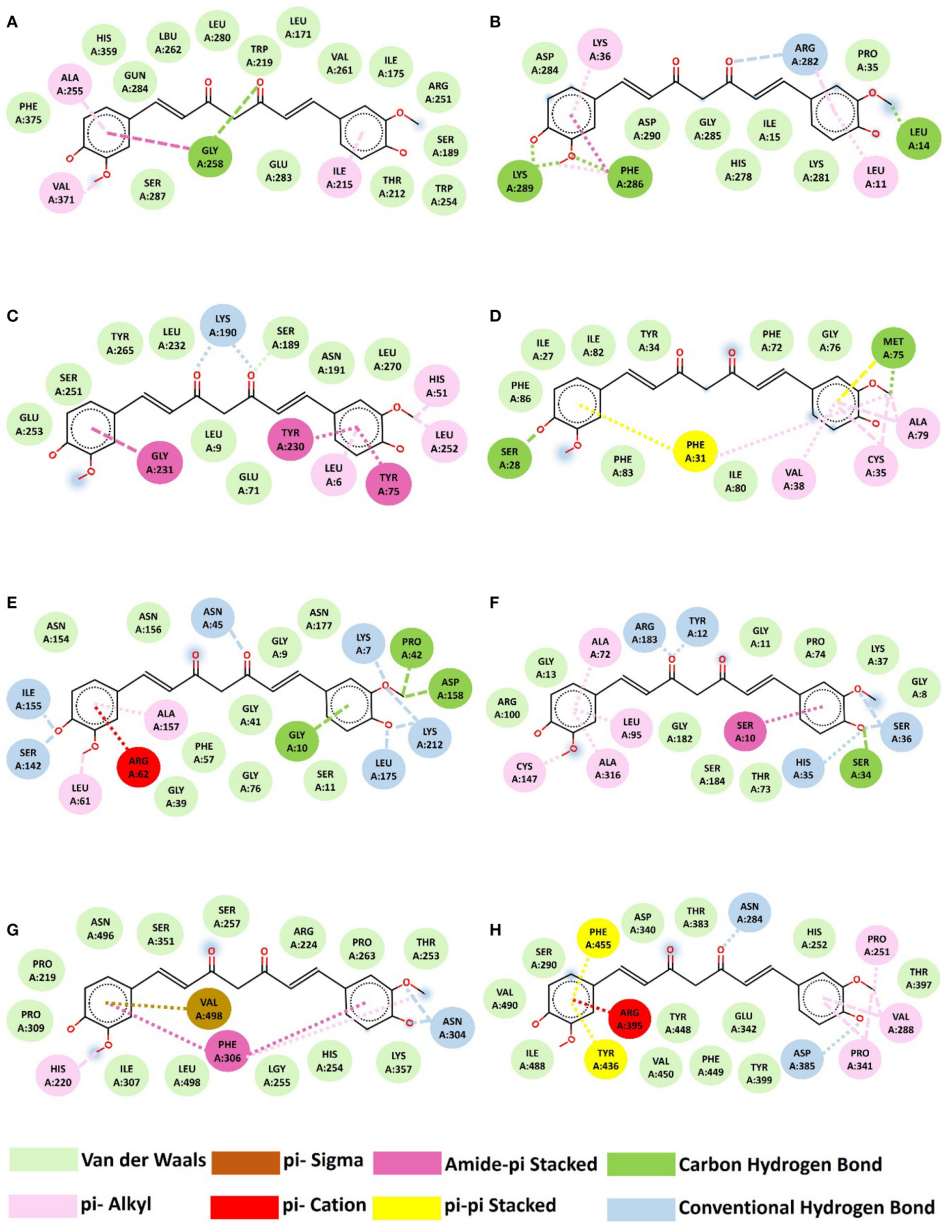
Interaction between curcumin and proteins. **(A)** *IcaA*-curcumin, **(B)** *IcaB*-curcumin, **(C)** *IcaC*-curcumin, **(D)** *IcaD*-curcumin, **(E)** *ArgB*-curcumin, **(F)** *ArgC*-curcumin, **(G)** *FnbA*-curcumin, **(H)** *CfbA*-Trrosseta *ClfA*.

Molecular docking algorithms recognize different ligand poses corresponding to different conformations and orientations within the chosen target binding site. They detect the preferred pose of curcumin inside a target binding site among these orientations using a scoring function that validates binding affinities and RMSD. We examined the highest binding affinity and ideal RMSD based on binding affinity < -5 kcal/mol and RMSD < 2 Å protocols. According to Docking's results (Table 2, Figure 4), there are different types of interactions between chosen targets and curcumin, such as H-bonding, hydrophobic, and van der Waals interactions. All of the interactions consist of H-bonding. H-bonding has the main role of promoting curcumin-binding affinity by displacing protein-bound water molecules into the bulk solvent. Furthermore, the reason for several hydrophobic interactions is the stabilization of the aromatic rings of curcumin. The highest binding affinity (−8.3 kcal/mol) with ideal RMSD belonged to *IcaD*, in which VAL38, ALA79, CYS35, MET75, PHE31, and SER28 residues were involved in the interactions between curcumin and *IcaD*. Conversely, *IcaC* possessed the lowest binding affinity (-6.9 kcal/mol) with ideal RMSD with five possible binding sites (LEU14, LEU11, ARG282, LYS36, PHE286, and LYS289). Overall, based on a molecular-level analysis, curcumin plays a significant role in the inhibition of all selected targets, especially *IcaD*. For the confirmation of the mentioned analysis, the following experimental evaluations were conducted.

**Table 2:** Docking result of curcumin with proteins.

**Proteins**	**Binding affinity**	**RMSD lower bond-upper bond**	**Interaction amino acid residue**
*IcaB*	−7.7	0	HIS51
			LEU252
			TYR75
			TYR230
			LEU6
			GLY231
			LYS190
			SER189
*IcaC*	−6.9	0	LEU14
			LEU11
			ARG282
			LYS36
			PHE286
			LYS289
*ArgB*	−7.0	0	ASN45
			PRO42
			LYS7
			LYS212
			LEU175
			GLY10
			ALA157
			ARG62
			SER142
			LEU61
			ILE155
*ArgC*	−7.6	0	SER36
			SER34
			HIS35
			SER10
			TYR12
			AGR183
			CYS147
			ALA72
			ALA316
			LEU95
*FnbA*	−7.2	0	ASN304
			PHE306
			VAL256
			HIS220
*CfbA*	−7.8	0	PRO251
			ASP385
			VAL288
			THR397
			PRO341
			TYR436
			PHE455
			ARG395
			ASN284
*IcaA*	−8.1	0	ILE215
			GLY258
			VAL371
			ALA255
*IcaD*	−8.3	0	VAL38
			ALA79
			CYS35
			MET75
			PHE31
			SER28

### 3.11 Empiric confirmation of the molecular docking prediction

**Figure 5:**
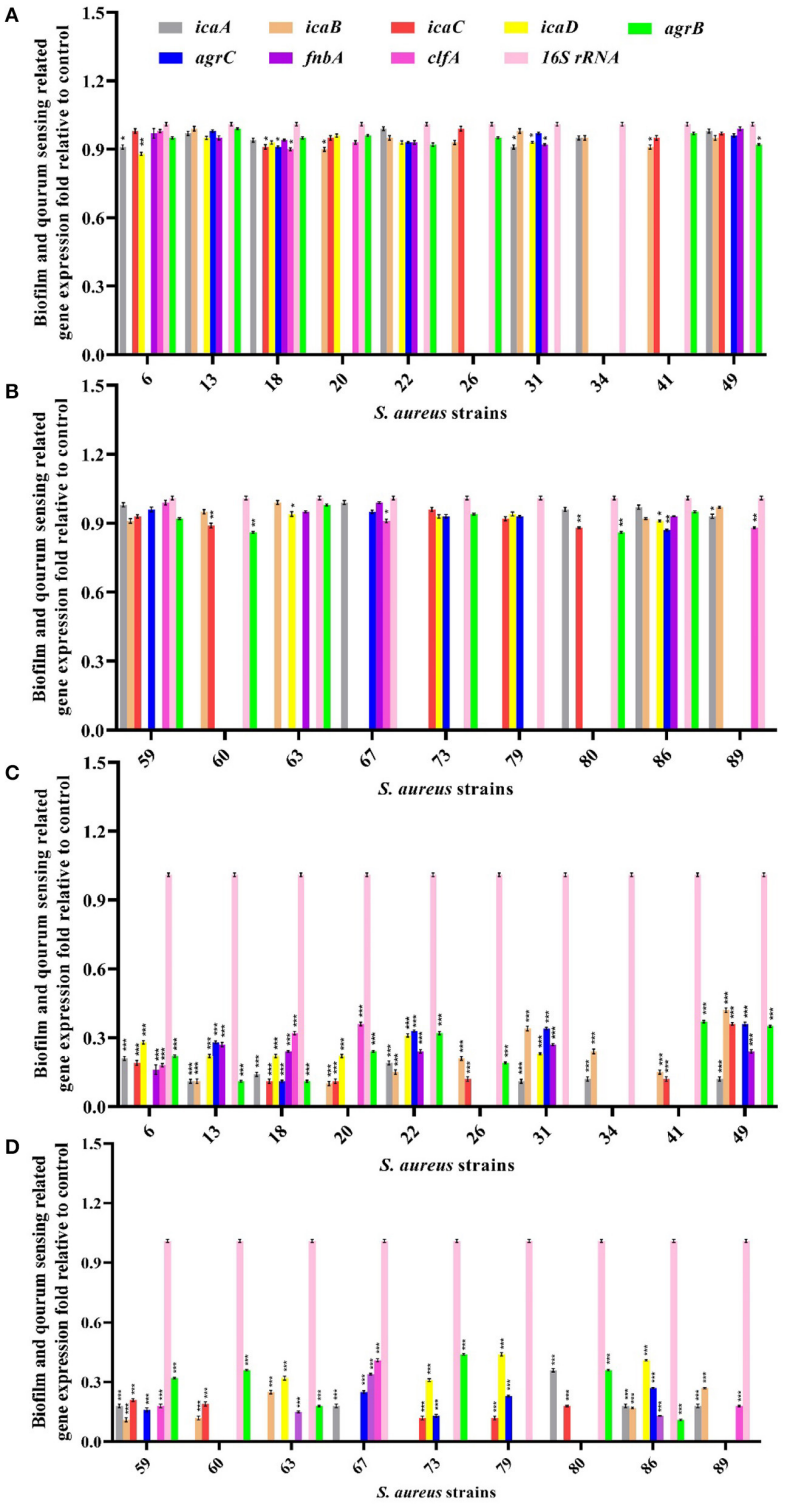
Evaluation of biofilm and quorum sensing-related gene expression in MRSA strains after treatment with half-MIC values of free curcumin **(A, B)** and its niosomal form **(C, D)**. ^***^p < 0.001, ^**^p < 0.01, and ^*^p < 0.05.

To investigate the perceived interaction between curcumin and biofilm-related proteins, biofilms treated with free curcumin and niosomal curcumin were analyzed for the transcription level of *icaA, icaB, icaC, icaD, fnbA, clfA, agrA, agrB*, and *agrC* genes using qRT-PCR. Our results demonstrated that the transcription level of the mentioned genes was downregulated significantly compared with free curcumin, indicating the anti-biofilm and anti-quorum sensing activity of niosome-encapsulated curcumin (Figure 5). One of the reasons for the decrease in the expression of genes involved in biofilm formation and quorum sensing can be the direct interaction between curcumin and transcription factors involved in biofilm (Piechota et al., 2018). Some studies showed that curcumin in MRSA strains could suppress the *mecA* gene expression, causing a decrease in the PBP2α protein level (Teow et al., 2016). MRSA in the presence of curcumin can, thus, be sensitive to β-lactam antibiotics, including penicillin and methicillin. It was also reported that curcumin shows a potent anti-biofilm effect by altering the quorum sensing gene expression and some virulence factors such as alginate production and swarming motility (Ghaffari et al., 2020). Some studies showed that curcumin has the potential for the downregulation of biofilm-related genes and also suppresses 31 quorum sensing gene expression and reduces virulence factors (Abdelbary et al., 2017). Another study demonstrated that curcumin has anti-infective activity by suppressing some biofilm and quorum-sensing genes in *Pseudomonas aeruginosa* (Shariati et al., 2019).

### 3.12 Cytotoxicity assay

**Figure 6:**
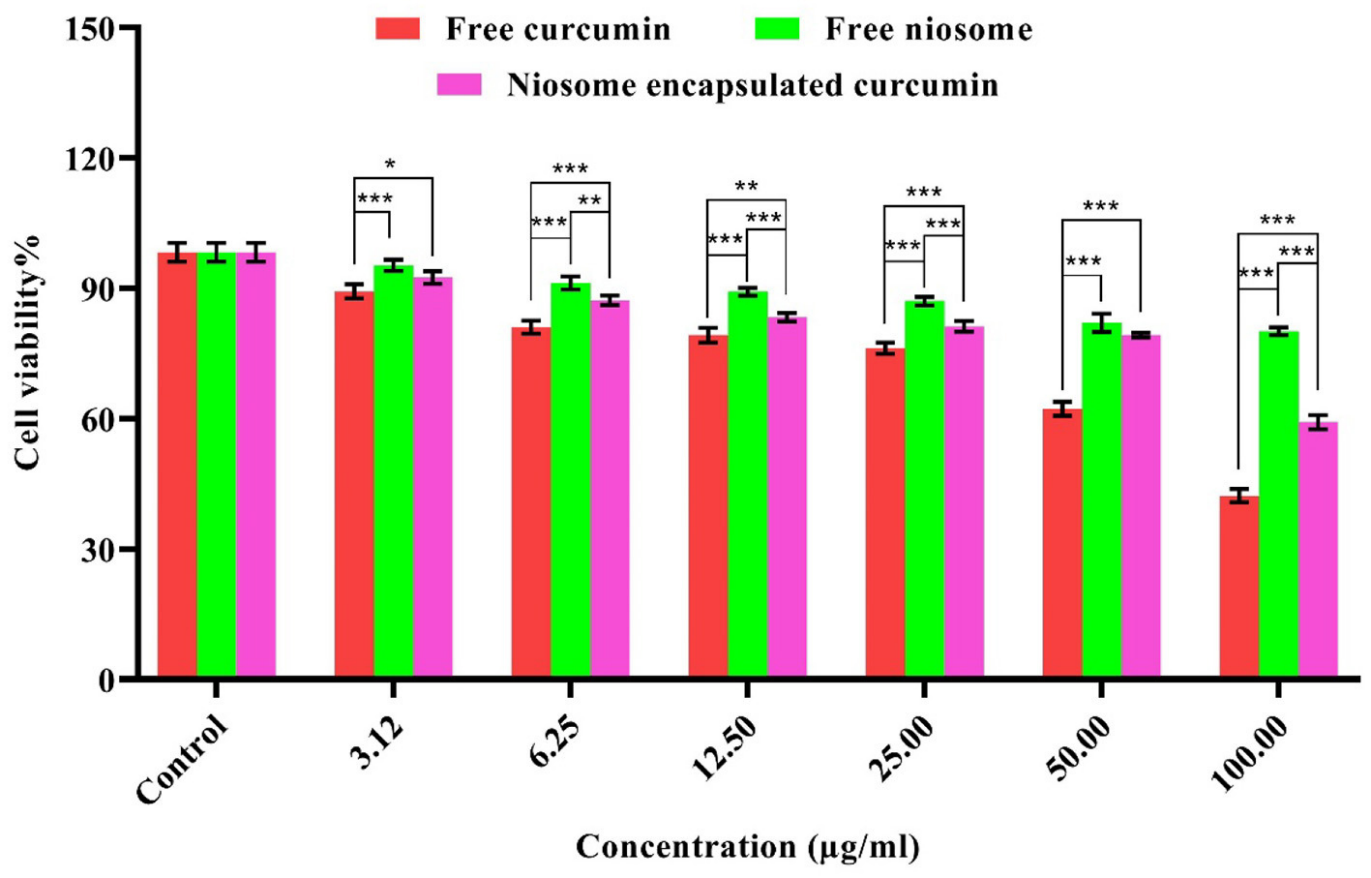
Cytotoxicity of free curcumin and niosome-encapsulated curcumin against HFF cells within 24 h. The results are shown in terms of cell survival (*p* < 0.001***, *p* < 0.01**, and *p* < 0.05*, *n* = 3).

The cytotoxicity was assessed *in vitro* for free niosome, free curcumin, and niosome-encapsulated curcumin using an MTT assay on a normal HFF cell line. Compared with the free curcumin, the curcumin encapsulated in the niosome showed less cytotoxic effect (Figure 6) and a much higher ratio of IC90 and MIC (IC90/MIC), i.e., the selectivity of the compound to inhibit the bacterium (Supplementary Table S2). Thus, niosomal curcumin is more biocompatible but more toxic to bacterial cells than free curcumin. Numerous studies show that free niosomes do not have significant cytotoxic effects on cells, indicating that niosomes are highly biocompatible and suitable for the drug delivery system (Xu et al., 2016; Akbarzadeh et al., 2021; Mousazadeh et al., 2022).

## 4 Conclusion

The present study aimed to use curcumin as an antimicrobial agent to fight against MRSA biofilms. Here, curcumin was analyzed for its suitability as a drug by ADME and consequently encapsulated with niosome as a drug delivery system. The niosomal formulations revealed more potential antibacterial and anti-biofilm activities against MDR *S. aureus* strains with negligible cytotoxicity against fibroblasts compared with the free curcumin. Molecular docking was employed to study the effect of curcumin on certain biofilm-relevant genes, which allowed us to interpret *in vitro* studies at the molecular level. The molecular docking analysis identified the significant role of *icaD* based on its highest binding affinity (-8.3) with ideal RMSD (0) and molecular interactions with curcumin. This prediction was confirmed by the empirical qRT-PCR results. Our findings suggest that niosome can provide a promising platform for the high efficiency of curcumin encapsulation and delivery and can be a suitable system for enhanced antibacterial and anti-biofilm activities against MDR *S. aureus* strains. Furthermore, computational methods can assist the mechanistic study of the drug and bacterial interactions and facilitate the identification of appropriate drug delivery systems.

## Data availability statement

The original contributions presented in the study are included in the article/Supplementary material, further inquiries can be directed to the corresponding authors.

## Author contributions

MK: Methodology, Writing – original draft. HS: Methodology, Writing – original draft. YH: Formal analysis, Methodology, Writing – review & editing. MM: Methodology, Writing – review & editing. ZM: Methodology, Writing – review & editing. BF: Formal analysis, Writing – review & editing. HN: Methodology, Writing – review & editing. AM: Conceptualization, Investigation, Supervision, Writing – review & editing. QR: Conceptualization, Supervision, Writing – review & editing.
