# Supplementary material for: In silico design and mechanistic study of niosome-encapsulated curcumin against multidrug-resistant Staphylococcus aureus biofilms
Source: Front Microbiol. 2023 Nov 30;14:1277533. doi: 10.3389/fmicb.2023.1277533 (PMC10720333; doi:10.3389/fmicb.2023.1277533)
Supplement: Supplementary file 1 [file Data_Sheet_1.pdf]

Supporting information:

In-silico design and mechanistic study of niosomes encapsulated curcumin against multidrug-resistant *Staphylococcus aureus* biofilms

Mohammad Khaleghian[1], Hamidreza Sahrayi[2], Yousef Hafezi[3], Mahshad Mirshafeeyan[2], Zahra Salehi Moghaddam[4], Bahareh Farasati Far[5], Hassan Noorbazargan[6], Amir Mirzaie[7, *], Qun Ren[8, *]

[1] Department of Chemistry, Payame Noor University, Tehran, Iran
[2] Department of Chemical and Petrochemical Engineering, Sharif University of Technology, Tehran, 1458889694, Iran
[3] School of Chemical Engineering, College of Engineering, University of Tehran, Tehran 14176, Iran
[4] Department of Microbial Biotechnology, School of Biology, College of Science, University of Tehran, 14155-6455 Tehran, Iran
[5] Department of Chemistry, Iran University of Science and Technology, Tehran, Iran
[6] Department of Biotechnology, School of Advanced Technologies in Medicine, Shahid Beheshti University of Medical Sciences, Tehran, Iran
[7] Department of Biology, Parand Branch, Islamic Azad University, Parand, Iran
[8] Laboratory for Biointerfaces, Empa, Swiss Federal Laboratories for Materials Science and Technology, 9014 St. Gallen, Switzerland

***Corresponding authors**

E-mail: amir_mirzaie92@yahoo.com (A. Mirzaie); Qun.Ren@empa.ch (Q. Ren)

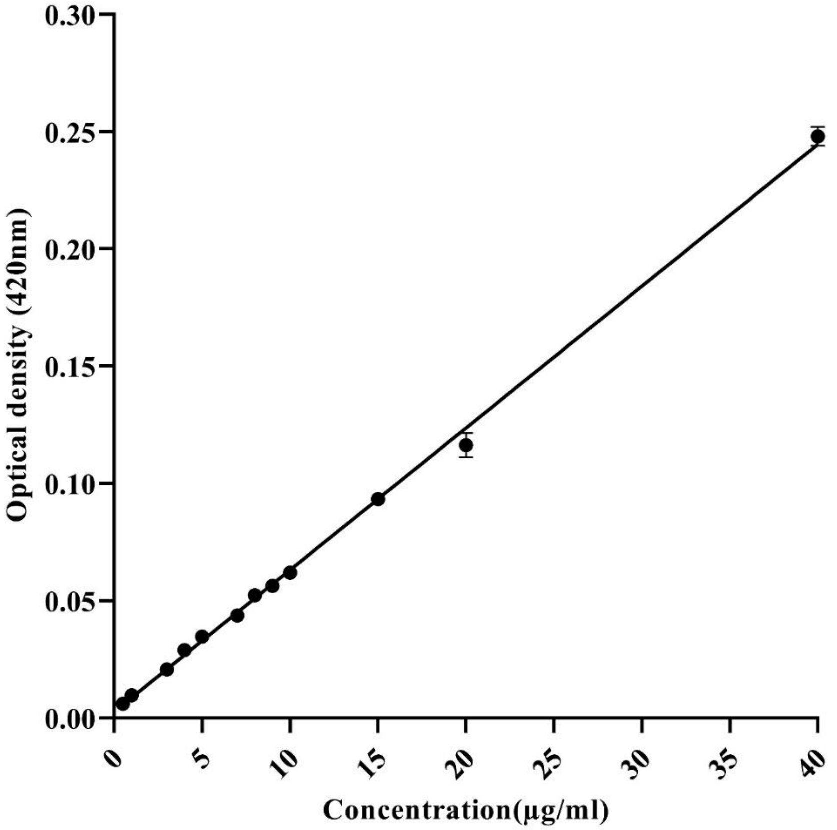

***Figure S1**: Calibration curve to determine the curcumin concentration using the absorbance reading at 420 nm.*

1. *In-silico* analysis

1.1. ADME analysis

In the early stages of drug discovery, ADME prediction is one of the imperative issues (Hadrup and Ravn-Haren, 2021). The software packages devoted to ADME assessment, ADMETLAB (http://admet.scbdd.com/calcpre/index/) and Swiss ADME (http://www.swissadme.ch/) determined Physico-chemical properties (Log S, log P, log D), Absorption(Caco-2 permeability, Human Intestinal Absorption, Pgp-inhibitor, and Pgp-substrate), Distribution(Plasma protein binding, Blood-brain barrier, Volume Distribution), Metabolism (CYPs), Excretion (Clearance, Half-life), Toxicity (hERG blockers, Human Hepatotoxicity). Furthermore, Lipinski's rule of five including Molecular weight, Lipophilicity (LogP), Hydrogen bond acceptor, and Hydrogen bond donors were evaluated (Verma and Thareja, 2017).

knowing more about the pharmacokinetics and pharmacodynamics features of curcumin can help to overcome these drawbacks. Here, 24 ADME features were checked based on ADMETLAB standards (**Figure S2**). In addition, to observe curcumin drug-likeness, Lipinski's rule-of-five proposes molecular weight ≤ 500, lipophilicity (LogP) ≤ 5, hydrogen bond acceptor ≤10, and hydrogen bond donors ≤ 5 (Hidayat et al., 2021). Curcumin has acceptable Lipinski's rule. The most important parameters of the curcumin for bioactivity depend on its physicochemical properties, including LogP, solubility (logS), and distribution coefficient (LogD). LogS, a measure of aqueous solubility, was found in the range of -4 to -6 which manifested moderate hydrophobicity. It is closely related to the LogP and logD features of curcumin which were higher than 3, and to the rarely investigated poor aqueous solubility and high membrane permeability. Compounds with slight solubility tend to have partially poor absorption, low stability, and fast clearance. According to the numerous clearance and Half-life of excretion part, curcumin revealed a rapid systemic clearance rate and very low half-life.

Among ADME features, caco-2 permeability, human intestinal absorption (HIA), and P-glycoprotein inhibitor and substrate are the most imperative desirable properties for curcumin absorption (Hou et al., 2007). High oral bioavailability is achieved from good oral absorption of HIA (Kosugi and Hosea, 2021). The most renewed human-intestinal cell is the Caco-2 cell, derived from colon carcinoma (Verhoeckx et al., 2015). Caco-2 cells express intestinal drug transporters therefore, they have a crucial role in gastrointestinal absorption (Shou, 2020). HIA and Caco-2 cells absorption cells of curcumin are appropriate. P-glycoprotein (P-gp) is one of the vital drug delivery barriers that transport back (efflux) toxins and xenobiotics from cells (Ghannay et al., 2020). According to the absorption chart and suitable ADMETLAB ranges, curcumin is not a P-gp substrate and inhibitor, therefore, it cannot transport back

to the plasma membrane or even induce drug resistance (Shawky et al., 2021). The curcumin uptake and distribution are impacted by plasma protein binding (PPB) (Wang et al., 2017). Curcumin has high PPB properties (approximately 87%). Since blood plasma consists of 92% of water, curcumin is hydrolyzed in aqueous plasma (Leung and Kee, 2009). It leads to a low therapeutic index with high degradation (Alqahtani et al., n.d.). It can certainly relate to the poor aqueous solubility of curcumin. Noureddin *et al.* reported that in human blood plasma, after 10 to 12 grams per day (high) oral administration of curcumin, the concentration of curcumin in plasma is in the nanomolar range. The low quantity of volume distribution indicated that curcumin was limited to blood plasma and bound to plasma protein.

The blood-brain barrier (BBB) is a collection of cell layers that connect the central nervous system (CNS) and the systemic circulation (Hettiarachchi and Leblanc, 2021). Some efflux transporters, including uptake transporters, are expressed by BBB, which can impact drug absorption in CNS (Wang and Hou, 2009). The curcumin penetration is 81%. Although curcumin can pass through from BBB, Low curcumin bioavailability and rapid elimination led to the failure of BBB clinical trials of curcumin (Chen et al., 2018). Yin-Meng *et al.* represented that nanoencapsulation of curcumin can influence on increasing time circulation of curcumin in the body (Tsai et al., 2011). The cytochrome P450s (CYPs) are crucial for ADME studies due to their key role in Phase I enzymes drug metabolism (Wang and Hou, 2009). Among 5 significant CYPs (CYPs 3A4, 2D6, 2C19, 2C9, and 1A2) responsible for drug biotransformation, curcumin-curcumin interactions are mediated by CYP2D6 inhibition leads to Drug adverse reactions and remain long after passes through the liver (Teschke and Danan, 2021). Drug metabolism through the CYP450 system has manifested as a significant factor in the appearance of drug-drug interactions that can end in toxicity. Curcumin human hepatotoxicity (H-HT) is one of the significant drawbacks. However, curcumin indicated a positive optimistic prediction for the human Ether-a-go-go Related Gene (hERG) potassium ion channel, which plays an important role in cardiotoxic effects via drug blockage (Sharma and Raghav, 2021).

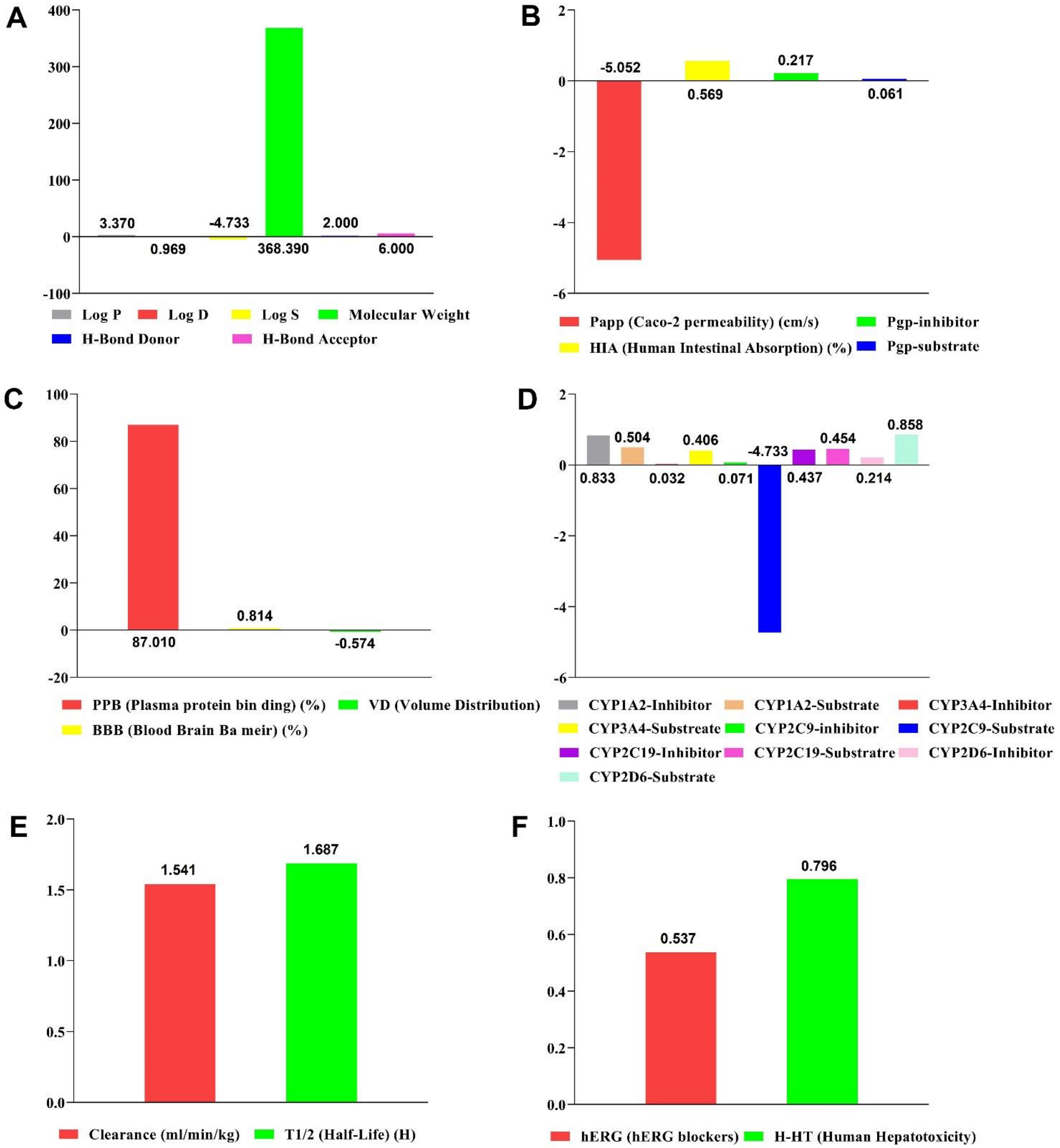

Figure S2: ADME prediction. **A)** Physico-chemical properties (LogP, logS, logD, Molecular weight, Hydrogen bond acceptor, and Hydrogen bond donors). **B)** Absorption (caco-2 permeability, HIA, and P-gp inhibitor and substrate). **C)** Distribution (PPB, BBB, and VD). **D)** Metabolism (CYPs). **E)** Excretion (clearance and half-life). **F)** Toxicity (hERG and H-HT).

1.2. Protein modeling and recognition of target properties

The purpose of the UniProt server is to provide a set of protein sequences with annotated functional properties (Consortium et al., 2021). All protein properties of IcaA (UniProt ID: A0A133Q3E9), (UniProt ID: A0A2X3QG95) IcaB, and IcaC (Uniprot ID: A0A2S6DQT9), IcaD (UniProt ID: A0A131JSY0), ArgB (UniProt ID: Q2G1H6), ArgC (Uniprot ID: A0A0D1HYU0), ClfA(Uniprot ID: Q2G015), FnbA(Uniprot ID: P14738) were retrieved from Uniprot server (https://www.uniprot.org/). Blastp databases found similar templates using high-scoring local alignments and the structure of proteins (IcaABCD, ArgB, ArgC, FnbA, clfA) were constructed from Swiss-Model Server (https://swissmodel.expasy.org/) based on homology modeling (Biasini et al., 2014). Model structures were compared with the models developed by the Robetta server (http://robetta.bakerlab.org/) and the trRosetta server (https://yanglab.nankai.edu.cn/trRosetta/) (Mahtarin et al., 2020). Moreover, the ProtParam server (https://web.expasy.org/protparam/) examined protein Physico-chemical features using the FASTA file (Sahay et al., 2020).

1.3. Validation of proteins

The quality evaluation analysis by the SAVES server (https://saves.mbi.ucla.edu/) reveals the excellent quality of the predicted models. The Ramachandran plot and ERRAT of the SAVES server were validated using the PDB file. Furthermore, The ProSA server (https://prosa.services.came.sbg.ac.at/) (Protein Structure Analysis) validates experimental and predicted protein structures.

1.4. Genes Refinement

Some of the proteins underwent refinement using GALAXYWEB server refinement (http://galaxy.seoklab.org/). The best-refined model was chosen based on the RMSD factor. Consequently, the refined structures compare with homology modeling structures. The final purpose was to select the better Ramachandran plot in which there aren't residues in the disallowed and generosity allowed.

1.5. Ligand preparation and molecular docking

Molecular docking mimics the biological environment and assesses the predominant interaction between ligand (Curcumin) and the three-dimensional structure of proteins(IcaA, IcaB, IcaC, IcaD, ArgC, ArgB, ClfA, FnbA) (Rahman et al., 2021). Curcumin's three-dimensional (3D) structures with SDF format were retrieved from the PubChem server (https://pubchem.ncbi.nlm.nih.gov/). UCSF Chimera v.1.8.1 software was used to remove co-crystallized ligands and solvents and the polar hydrogens were

added (Joshi et al., 2020). After preparing the protein's PDB structures as macromolecules in PyRx v.0.8 tools, Autodock Tools 1.5.6 software minimized Curcumin's energy and saved it as a PDBQT file. Autodock Vina 1.1.2 was used to dock Curcumin with proteins, respectively. the grid boxes of proteins were maximized. Binding affinity and RMSD play a significant role in generated conformers. Furthermore, 2D interactions of Curcumin and targeted proteins were visualized using Discovery Studio 3.1 software.

Table S1: Different levels were used to optimize the variables in the Box-Behnken test.

Level	-1	0	+1
A (Drug concentration, mg/ml)	0.5	1	1.5
B (Surfactant to Cholesterol, molar ratio)	0.5	1	2.0
C (Lipid to Drug, molar ratio)	10.0	20	30.0

Table S2: The MIC values and selectivity of free curcumin and niosome encapsulated curcumin against biofilm-forming MDR-MRSA strains.

MRSA Strain NO.	MIC values of free curcumin (μg/ml)	Selectivity (IC_{90}/MIC)	MIC values of niosome encapsulated curcumin (μg/ml)	Selectivity (IC_{90}/MIC)
6	125±0.0	1.447±0.0	15.62±0.0	14.266±0.0
13	250±0.0	0.724±0.0	31.25±0.0	7.131±0.0
20	250±0.0	0.724±0.0	62.50±0.0	3.565±0.0
22	125±0.0	1.447±0.0	31.25±0.0	7.131±0.0
26	250±0.0	0.724±0.0	31.25±0.0	7.131±0.0
28	250±0.0	0.724±0.0	31.25±0.0	7.131±0.0
31	125±0.0	1.447±0.0	15.62±0.0	14.266±0.0
34	125±0.0	1.447±0.0	15.62±0.0	14.266±0.0
41	250±0.0	0.724±0.0	31.25±0.0	7.131±0.0
49	125±0.0	1.447±0.0	15.62±0.0	14.266±0.0
54	250±0.0	0.724±0.0	62.50±0.0	3.565±0.0
59	125±0.0	1.447±0.0	15.62±0.0	14.266±0.0
60	125±0.0	1.447±0.0	15.62±0.0	14.266±0.0
63	125±0.0	1.447±0.0	31.25±0.0	7.131±0.0
67	250±0.0	0.724±0.0	31.25±0.0	7.131±0.0
73	125±0.0	1.447±0.0	15.62±0.0	14.266±0.0
79	250±0.0	0.724±0.0	31.25±0.0	7.131±0.0
80	125±0.0	1.447±0.0	15.62±0.0	14.266±0.0
86	250±0.0	0.724±0.0	31.25±0.0	7.131±0.0
89	125±0.0	1.447±0.0	15.62±0.0	14.266±0.0

Table S3: Experimental design using the Box-Benken method to optimize the different formulations of niosome encapsulated curcumin. A: drug concentration, B: molar ratio of surfactant:cholesterol, C: molar ratio of lipid:drug.

**Run**	**Levels of independent variables**			**Dependent variables**		
	A (mg/ml)	B (mol ratio)	C (mol ratio)	Size (nm)	Polydispersity Index (PDI)	EE (%)
1	0	-1	-1	269	0.30	80
2	0	0	0	191	0.27	95
3	0	1	-1	199	0.31	84
4	-1	0	-1	163	0.29	86
5	0	1	1	296	0.31	94
6	1	1	0	245	0.21	91
7	1	0	1	281	0.29	97
8	0	-1	1	358	0.24	92
9	-1	1	0	224	0.25	90
10	-1	-1	0	306	0.31	87
11	0	0	0	187	0.28	96
12	1	0	-1	172	0.25	97
13	1	-1	0	322	0.29	89
14	-1	0	1	209	0.26	94
15	0	0	0	187	0.28	95

Table S4: Analysis of variance for the quadratic polynomial model for size.

Source	Sum of squares	Degree of Freedom	Mean square	F-Value	P-Value	Evaluation
Model	51617.35	9	5735.26	60.90	0.0001	Significant
A	1743.45	1	1743.45	18.51	0.0077	Significant
B	10679.91	1	10679.91	113.41	0.0001	Significant
C	14433.00	1	14433.00	153.27	< 0.0001	Significant
AB	4.41	1	4.41	0.047	0.8372	
AC	1001.72	1	1001.72	10.64	0.0224	Significant
BC	14.82	1	14.82	0.16	0.7079	
A^2	118.22	1	118.22	1.26	0.3134	
B^2	23606.16	1	23606.16	250.68	< 0.0001	Significant
C^2	539.10	1	539.10	5.72	0.0622	
Residual	470.85	5	94.17			Significant

Table S5: Analysis of variance for the quadratic polynomial model for EE[1]

Source	Sum of squares	Degree of Freedom	Mean square	F-Value	P-Value	Evaluation
Model	317.09	9	317.09	5.24	0.0415	Significant
A	34.24	1	34.24	5.39	0.0397	Significant
B	11.76	1	11.76	1.75	0.2434	
C	109.15	1	109.15	16.22	0.0100	Significant
AB	0.86	1	0.86	0.13	0.7359	
AC	11.02	1	11.02	1.64	0.2567	
BC	1.68	1	1.68	0.25	0.6388	
A^2	0.054	1	0.054	8.012E-003	0.9321	
B^2	138.67	1	138.67	20.61	0.0062	Significant
C^2	15.08	1	15.08	2.24	0.1946	
Residual	33.64	5	33.64			

[1]$EE = 95.82 + 2.07\times A + 2.21\times B + 3.69\times C - 0.46\times A\times B - 1.66\times A\times C - 0.65\times B\times C - 0.12\times A^2 - 6.13\times B^2 - 2.02\times C^2$

Table S6: Predicted values and desirability criteria obtained for variables.

Number	A	B	C	Desirability
1	1	1:1	12:1	0.926

Table S7: The results obtained using the method and its comparison with the results of experimental tests.

Parameter	Predicted by RSM	Experimental Data
Average size (nm)	163	177±4
Encapsulation Efficiency (EE) (%)	94	97±1

2. Kinetic release models

The explanation of each kinetic model used in this study is as follows:

2.1. Zero-order model: $\boldsymbol{C_t = C_0 + K_0 t}$

where C_t represents the amount of drug released at time t, and C_0 is the initial concentration of drug released which is generally zero. In this model, the release process takes place at a constant rate and is independent of the initial drug concentration.

2.2. First-order model: $\boldsymbol{Log\ C = LogC_0 + Kt}$

Where C_0 is the initial concentration of the drug, k is the first-order rate constant, and t is the time. C is the drug remaining in the carrier at time t. Log C and t have s linear relationship and K/2.303 is the slope of the straight line. This model can be used to describe water-soluble drugs in porous matrices.

2.3. Higuchi model: $\boldsymbol{Q = Q_0 + K_H \sqrt{t}}$

where K_H is the Higuchi constant and it is obtained from the slope of the line. The data obtained were plotted as cumulative percentage drug release versus square root of time. This model can be useful in the case of matrix tablets containing water-soluble drugs.

2.4. Korsmeyer-Peppas model: $\boldsymbol{M_t / M_\infty = Kt^n}$

Linear Form: $\boldsymbol{\log M = \log K + n \log t}$

where M_t/M_0 is a fraction of the drug released at time t, k is the release rate constant and n is the release exponent. The n value is used to characterize different releases for cylindrical-shaped matrices. In the case of spherical tablets:

n ≤ 0.43: Fickian diffusion mechanism

0.43 < n <0.85: non-Fickian transport.

n = 0.85: Case II (relaxational) transport.

n > 0.85: super case II transport.

Table S8: FTIR spectra in curcumin and niosome samples containing curcumin.

Sample, chemicals	Peak cm^{-1}	Description
DCP	1243	P=O bonding
	724-843	P-O bonding
	2965	C-H stretching
	1450	CH3 bonding
	1465	CH2 bonding
Span 80	1000-1300	C–O stretching
	2800-3000	C-H stretching
	3452	OH stretching
	1749	CH2 bonding
	1753	C =O stretching
	1497	Aromatic ring
Cholesterol	1747	C= O stretching
	2800-3000	C-H stretching
	3452	OH stretching
	1035-1378	CH_2 bending and CH_2 deformation
	1506	C-C stretching in the aromatic ring
	1674	C=C stretching
Niosome	1165	C–O stretching
	1745	C = O stretching
	2800-3000	C-H stretching
	1498	Aromatic ring
	1000-1250	Aliphatic C-N stretching
	3452	OH stretching
Curcumin	1148	C=O stretch
	600-800	C–H out-of-plane bending vibrations
	3517	C-H stretching and O–H stretching
	3452	OH stretching
	1523	aromatic ring C=C stretching
curcumin -loaded Niosome	1125	C–O stretching
	1747	C = O stretching
	2800-3000	C-H stretching
	3452	OH stretching
	1000-1250	Aliphatic C-N stretching
	2240	C≡N stretching

Table S9: Kinetic models obtained from optimally synthesized niosomes as well as drug release patterns.

Release Model	Zero-Order	Korsmeyer-Peppas		First-Order	Higuchi
	R^2	R^2	N	R^2	R^2
Free Curcumin-pH 7.4 (A)	0.3903	0.5892	0.5433	0.7787	0.5992
Curcumin loaded niosome- pH 7.4 (D)	0.8297	0.9526	0.4292	0.8969	0.9459
Curcumin loaded niosome- pH 5 (C)	0.8575	0.9754	0.6863	0.9459	0.9114
Curcumin loaded niosome- pH 3 (B)	0.6075	0.9353	0.6324	0.8526	0.6075

* Diffusion or release exponent

Table S10: The antibiotic resistance pattern of MDR *S. aureus* strains

Strain NO.	Antibiotic resistance pattern
6	PEN, OXA, CEF, AMI
13	OXA, CEF, GEN
20	OXA, CEF, AMO, GEN
22	OXA, CEF, AMI, TRI, ERY
26	OXA, CEF, AMO
31	OXA, CEF, PEN, AMI
34	OXA, CEF, AMI
41	OXA, CEF, CHL, GEN
49	OXA, CEF, AMO, AMI
59	OXA, CEF, CHL, AMO, AMI
60	OXA, CEF, AMO, GEN
63	OXA, CEF, AMO, AMI
67	OXA, CEF, AMI
73	OXA, CEF, ERY, GEN
79	OXA, CEF, GEN
80	OXA, CEF, ERY, AMO, GEN
86	OXA, CEF, AMO, TRI, AMI
89	OXA, CEF, AMO, AMI

PEN: Penicillin, OXA: Oxacillin, CEF: Ceftriaxone, AMI: Amikacin, GEN: Gentamycin, TRI: Trimethoprim, ERY: Erythromycin, CHL: Chloramphenicol, AMO: Amoxicillin

Table S11: The MIC values of free curcumin and niosome encapsulated curcumin against some Gram-positive and Gram-negative standard strains.

**Bacteria**	**MIC values of free curcumin (µg/ml)**	**MIC values of niosome encapsulated curcumin (µg/ml)**
Escherichia coli ATCC 25922	250±0.0	31.25±0.0
Pseudomonas aeruginosa ATCC 27853	250±0.0	31.25±0.0
Klebsiella pneumoniae ATCC 700603	500±0.0	62.5±0.0
Proteus mirabilis ATCC 12453	250±0.0	31.25±0.0
Staphylococcus aureus ATCC 700698	125±0.0	15.62±0.0
Bacillus subtilis ATCC 21332	250±0.0	31.25±0.0
Streptococcus pyogenes ATCC 14918	125±0.0	15.62±0.0
Staphylococcus epidermidis ATCC 12228	125±0.0	15.62±0.0

References

Alqahtani, A., Chidambaram, K. Pino-Figueroa, K., Chandrasekaran, B., Dhanaraj, P., Venkatesan, P. (2021). Curcumin-Celecoxib: a synergistic and rationale combination chemotherapy for breast cancer. *Eur. Rev. Med. Pharmacol. Sci.* doi: 10.26355/eurrev_202102_25086.

Biasini, M., Bienert, S., Waterhouse, A., Arnold, K., Studer, G., Schmidt, T., et al. (2014). SWISS-MODEL: modelling protein tertiary and quaternary structure using evolutionary information. *Nucleic Acids Res.* 42, W252–W258. doi: 10.1093/NAR/GKU340.

Chen, M., Du, Z. Y., Zheng, X., Li, D. L., Zhou, R. P., and Zhang, K. (2018). Use of curcumin in diagnosis, prevention, and treatment of Alzheimer's disease. *Neural Regen. Res.* 13. doi: 10.4103/1673-5374.230303.

Consortium, T. U., Bateman, A., Martin, M.-J., Orchard, S., Magrane, M., Agivetova, R., et al. (2021). UniProt: the universal protein knowledgebase in 2021. *Nucleic Acids Res.* 49, D480–D489. doi: 10.1093/NAR/GKAA1100.

Ghannay, S., Kadri, A., and Aouadi, K. (2020). Synthesis, in vitro antimicrobial assessment, and computational investigation of pharmacokinetic and bioactivity properties of novel trifluoromethylated compounds using in silico ADME and toxicity prediction tools. *Monatshefte fur Chemie* 151, 267–280. doi: 10.1007/S00706-020-02550-4.

Hadrup, N., and Ravn-Haren, G. (2021). Absorption, distribution, metabolism and excretion (ADME) of oral selenium from organic and inorganic sources: A review. *J. Trace Elem. Med. Biol.* 67, 126801. doi: 10.1016/J.JTEMB.2021.126801.

Hettiarachchi, S., and Leblanc, R. (2021). Dual targeting nano-approaches for Alzheimer's disease etiology. *Neural Regen. Res.* 16, 119. doi: 10.4103/1673-5374.286965.

Hidayat, S., Ibrahim, F., Pratama, K., and Muchtaridi, M. (2021). The interaction of alpha-mangostin and its derivatives against main protease enzyme in COVID-19 using in silico methods. *J. Adv. Pharm. Technol. Res.* 12, 285. doi: 10.4103/JAPTR.JAPTR_299_20.

Hou, T., Wang, J., Zhang, W., and Xu, X. (2007). ADME evaluation in drug discovery. 7. prediction of oral absorption by correlation and classification. *J. Chem. Inf. Model.* 47, 208–218. doi: 10.1021/CI600343X.

T Joshi, T., Joshi, T., Sharma, P., Mathpal, S., Pundir, H., Bhatt, V., Chandra, S. (2020). In silico screening of natural compounds against COVID-19 by targeting Mpro and ACE2 using molecular docking. *Eur Rev Med Pharmacol Sci*. doi: 10.26355/eurrev_202004_21036.

Kosugi, Y., and Hosea, N. (2021). Prediction of Oral Pharmacokinetics Using a Combination of in Silico Descriptors and in Vitro ADME Properties. *Mol. Pharm.* 18, 1071–1079. doi: 10.1021/ACS.MOLPHARMACEUT.0C01009.

Leung, M. H. M., and Kee, T. W. (2009). Effective stabilization of curcumin by association to plasma proteins: Human serum albumin and fibrinogen. *Langmuir* 25, 5773–5777. doi: 10.1021/LA804215V.

Mahtarin, R., Islam, S., Islam, M. J., Ullah, M. O., Ali, M. A., and Halim, M. A. (2020). Structure and dynamics of membrane protein in SARS-CoV-2. *J. Biomol. Struct. Dyn.* doi: 10.1080/07391102.2020.1861983.

Rahman, F., Tabrez, S., Ali, R., Alqahtani, A. S., Ahmed, M. Z., and Rub, A. (2021). Molecular docking analysis of rutin reveals possible inhibition of SARS-CoV-2 vital proteins. *J. Tradit. Complement. Med.* 11, 173–179. doi: 10.1016/J.JTCME.2021.01.006.

Sahay, A., Piprodhe, A., and Pise, M. (2020). In silico analysis and homology modeling of strictosidine synthase involved in alkaloid biosynthesis in catharanthus roseus. *J. Genet. Eng. Biotechnol.* 18. doi: 10.1186/S43141-020-00049-3.

Sharma, K., and Raghav, N. (2021). Curcumin analogs as anti-cathepsins agents: Designing, virtual screening,

and molecular docking analysis. *Comput. Toxicol.* 19, 100174. doi: 10.1016/J.COMTOX.2021.100174.

Shawky, A. M., Ibrahim, N. A., Abourehab, M. A. S., Abdalla, A. N., and Gouda, A. M. (2021). Pharmacophore-based virtual screening, synthesis, biological evaluation, and molecular docking study of novel pyrrolizines bearing urea/thiourea moieties with potential cytotoxicity and CDK inhibitory activities. *J. Enzyme Inhib. Med. Chem.* 36, 15–33. doi: 10.1080/14756366.2020.1837124.

Shou, W. Z. (2020). Current status and future directions of high-throughput ADME screening in drug discovery. *J. Pharm. Anal.* 10, 201–208. doi: 10.1016/J.JPHA.2020.05.004.

Teschke, R., and Danan, G. (2021). Idiosyncratic Drug Induced Liver Injury, Cytochrome P450, Metabolic Risk Factors and Lipophilicity: Highlights and Controversies. *Int. J. Mol. Sci. 2021, Vol. 22, Page 3441* 22, 3441. doi: 10.3390/IJMS22073441.

Tsai, Y. M., Chien, C. F., Lin, L. C., and Tsai, T. H. (2011). Curcumin and its nano-formulation: The kinetics of tissue distribution and blood-brain barrier penetration. *Int. J. Pharm.* 416. doi: 10.1016/j.ijpharm.2011.06.030.

Verhoeckx, K., Cotter, P., López-Expósito, I., and Kleiveland, C. (2015). The impact of food bioactives on health: in vitro and ex vivo models. Available at: https://library.oapen.org/bitstream/handle/20.500.12657/28028/1001968.pdf?sequence=1 [Accessed September 22, 2021].

Verma, S. K., and Thareja, S. (2017). Structure based comprehensive modelling, spatial fingerprints mapping and ADME screening of curcumin analogues as novel ALR2 inhibitors. *PLoS One* 12. doi: 10.1371/JOURNAL.PONE.0175318.

Wang, J., and Hou, T. (2009). Chapter 5 Recent Advances on in silico ADME Modeling. *Annu. Rep. Comput. Chem.* 5, 101–127. doi: 10.1016/S1574-1400(09)00505-2.

Wang, N. N., Deng, Z. K., Huang, C., Dong, J., Zhu, M. F., Yao, Z. J., et al. (2017). ADME properties evaluation in drug discovery: Prediction of plasma protein binding using NSGA-II combining PLS and consensus modeling. *Chemom. Intell. Lab. Syst.* 170, 84–95. doi: 10.1016/J.CHEMOLAB.2017.09.005.
